# Evaluation of Scalable Synthesis Methods for Aluminum-Substituted Li_7_La_3_Zr_2_O_12_ Solid Electrolytes

**DOI:** 10.3390/ma14226809

**Published:** 2021-11-11

**Authors:** Markus Mann, Michael Küpers, Grit Häuschen, Martin Finsterbusch, Dina Fattakhova-Rohlfing, Olivier Guillon

**Affiliations:** 1 Institute of Energy and Climate Research (IEK-1) Materials Synthesis and Processing, Forschungszentrum Jülich GmbH, Wilhelm-Johnen-Strasse, 52425 Jülich, Germany; m.mann@fz-juelich.de (M.M.); m.kuepers@fz-juelich.de (M.K.); g.haeuschen@fz-juelich.de (G.H.); d.fattakhova@fz-juelich.de (D.F.-R.); o.guillon@fz-juelich.de (O.G.); 2 Helmholtz-Institut Münster (IEK-12), Forschungszentrum Jülich GmbH, Corrensstraße 46, 48149 Münster, Germany; 3 Jülich Aachen Research Alliance, JARA-Energy, 52425 Jülich, Germany

**Keywords:** all-solid-state battery, ceramic solid electrolyte, LLZO, scale-up

## Abstract

Solid electrolyte is the key component in all-solid-state batteries (ASBs). It is required in electrodes to enhance Li-conductivity and can be directly used as a separator. With its high Li-conductivity and chemical stability towards metallic lithium, lithium-stuffed garnet material Li_7_La_3_Zr_2_O_12_ (LLZO) is considered one of the most promising solid electrolyte materials for high-energy ceramic ASBs. However, in order to obtain high conductivities, rare-earth elements such as tantalum or niobium are used to stabilize the highly conductive cubic phase. This stabilization can also be obtained via high levels of aluminum, reducing the cost of LLZO but also reducing processability and the Li-conductivity. To find the sweet spot for a potential market introduction of garnet-based solid-state batteries, scalable and industrially usable syntheses of LLZO with high processability and good conductivity are indispensable. In this study, four different synthesis methods (solid-state reaction (SSR), solution-assisted solid-state reaction (SASSR), co-precipitation (CP), and spray-drying (SD)) were used and compared for the synthesis of aluminum-substituted LLZO (Al:LLZO, Li_6.4_Al_0.2_La_3_Zr_2_O_12_), focusing on electrochemical performance on the one hand and scalability and environmental footprint on the other hand. The synthesis was successful via all four methods, resulting in a Li-ion conductivity of 2.0–3.3 × 10^−4^ S/cm. By using wet-chemical synthesis methods, the calcination time could be reduced from two calcination steps for 20 h at 850 °C and 1000 °C to only 1 h at 1000 °C for the spray-drying method. We were able to scale the synthesis up to a kg-scale and show the potential of the different synthesis methods for mass production.

## 1. Introduction

Due to their high energy density and cycle stability, lithium-ion batteries (LIB) are one of the most common battery types in mobile and stationary applications today [1,2]. However, after almost 30 years of development and optimization since their market launch, LIBs are about to reach their physicochemical limit [3]. For even higher energy densities, new battery concepts are currently being developed which could theoretically outperform conventional LIBs. Advanced battery concepts such as all-solid-state batteries (ASB) are considered as one of the most promising candidates for future energy storage technologies. They offer several advantages over conventional LIBs with regard to stability, safety, and energy density [4,5]. Especially in regard to safety (flammability and toxicity), ASBs based on ceramic electrolytes are not surpassed by any other class of materials [6]. Due to its high Li-ion conductivity (up to 1 × 10^−3^ S/cm), garnet-based Li_7_Zr_3_La_2_O_12_ compounds are considered the most promising for oxide-based ceramic electrolyte ASSB. Its stability towards metallic lithium allows the direct use of lithium as an anode material without any further stabilization [7,8]. To stabilize the high lithium-ion conducting cubic garnet phase at room temperature, substitution of Li_7_Zr_3_La_2_O_12_ is necessary. Several substituents were investigated in the literature, such as Al, Ta, Ga, Te, W, Fe, or Nb [9,10,11,12,13,14]. Thus far, the highest conductivities were reached using tantalum (1.35 × 10^−3^ S/cm) [15] and gallium (1.84 × 10^−3^ S/cm) [16]. However, as the required substitution levels and their prices are rather high, they also make the material more expensive.

Depending on the application, a balancing between conductivity and price needs to be carefully evaluated. High Li-ion conductivity is crucial for mobile devices, especially in the automotive sector, where fast charging and power densities are important. For other applications, for example, stationary storage, other properties are more crucial, such as safety aspects or price per kWh stored. Especially with respect to cost-effectiveness, Al-doped LLZO is an interesting candidate. Aluminum was one of the first substituents for LLZO that was investigated, initially by accident, since the LLZO samples were sintered in Al_2_O_3_-crucibles, later on purpose, forming the highly conductive cubic garnet structure [13,17]. The highest total Li-ion conductivity reached for Al:LLZO was 6.8 × 10^−4^ S/cm [18]. Since the discovery of highly conductive LLZO, various synthesis routes were investigated to synthesize Al:LLZO, with a classical solid-state reaction being the most common one [11,13,19]. In addition, wet-chemical methods such as sol-gel [20], Pechini [21], nebulized spray pyrolysis [22], chemical co-precipitation [23], or combustion [24] were shown to be suitable for the synthesis of Al-doped cubic LLZO. Comparing the results of the different methods is hard since they not only differ in the obtained particle size but also in stoichiometry (especially Al-content) and sintering conditions to obtain the samples for conductivity measurements. Table 1 shows total conductivities and sintering conditions for some selected Al:LLZO, ranging between 10^−6^ and 10^−4^ S/cm. It has to be noted that some of the synthesis methods require high amounts of solvents or additives (e.g., the sol-gel and combustion methods), making them unattractive for industrial up-scaling.

In this work, we synthesized Al:LLZO (Li_6.4_Al_0.2_La_3_Zr_2_O_12_) via four different synthesis routes with a focus on scalability: solid-state reaction (SSR), solution-assisted solid-state reaction (SASSR), spray-drying (SD), and co-precipitation (CP). We only used water as the solvent and omitted synthesis routes that require additional additives such as sol-gel and the combustion method. Keeping the stoichiometry and sintering conditions constant allows us to evaluate the impact of the different synthesis routes on the electrochemical performance and enables us to evaluate scalability, cost, and industrial applicability.

## 2. Materials and Methods

### 2.1. Synthesis

#### 2.1.1. Solid-State Reaction

For the solid-state reaction, the starting materials LiOH·H_2_O (99%, AppliChem GmbH, Darmstadt, Germany), La_2_O_3_ (99.9%, Merck KGaA, Darmstadt, Germany, 10 h pre-dried at 900 °C), ZrO_2_ (99.5%, Alfa Aesar GmbH & Co KG, Karlsruhe, Germany), and Al_2_O_3_ (99.9%, Inframat Advanced Materials LLC, Manchester, CT, USA) were weighed stoichiometrically in 100 g batches and ground with an electrical mortar grinder (RM 200, Retsch GmbH, Haan, Germany) for 1 h. From the homogenized powder, pellets were pressed (uniaxial, 45 mm diameter, at 20 MPa) and calcined twice for 20 h in alumina crucibles. The first calcination step was performed at 850 °C, whilst the second one was performed at 1000 °C. After each calcination step, the pellets were ground to powder and repressed to pellets.

#### 2.1.2. Solution-Assisted Solid-State Reaction

In the solution-assisted solid-state reaction, all starting materials Al(NO_3_)_3_·9H_2_O (>98%, Merck KGaA, Darmstadt, Germany), LiNO_3_ (99%, Alfa Aesar GmbH & Co KG, Karlsruhe, Germany), and La(NO_3_)_3_·6H_2_O (99.99%, chemPUR Feinchemikalien und Forschungsbedarf GmbH, Karlsruhe, Germany) were dissolved in H_2_O. ZrO(NO_3_)_2_·*x*H_2_O (99%, Merck KGaA, Darmstadt, Germany) was dissolved in a nitric acid solution while stirring at 60 °C. The exact Zr-concentration was determined by ICP-OES. The solutions were mixed stoichiometrically, and the water was evaporated while stirring at 150 °C on a magnetic heat stirrer. The resulting powder was finally dried in a drying chamber at 95 °C for seven days. The dry powder was then calcined at 800 °C for 1 h to decompose nitrates and hydroxides. After calcination, the powder was mortared in an electrical mortar grinder for 1 h and recalcined at 1000 °C for 20 h, followed by an additional mortaring step.

#### 2.1.3. Co-Precipitation

The starting materials of La-nitrate, Zr-nitrate, and Al-nitrate were dissolved in H_2_O/nitric acid as described above. The acidic solution was then dropped into a NH_4_OH-basic solution with a pH-value of 9.5 at 60 °C. The pH-value was monitored throughout the precipitation and adjusted by adding aqueous NH_4_OH- and LiOH-solution. A white precipitate was formed, which was filtered and dried in a drying chamber at 95 °C for seven days. The dried powder was ground in a mortar together with a stoichiometric amount of LiOH (10% excess). It was then calcined at 1000 °C for 1 h and subsequently ground in an electrical mortar to break up agglomerates.

#### 2.1.4. Spray-Drying

The same precursor solution was used as described in the SASSR above. The solution was sprayed into 300 °C hot air in a pilot plant spray dryer (Nubilosa, Konstanz, Germany). This results in strongly hygroscopic white powder, which was calcinated at 1000 °C for 1 h and finally ground using an electrical mortar.

#### 2.1.5. Sintering

For better comparison, the sintering conditions were kept the same for all syntheses. For each sample, 7 g of the finely ground powder was uniaxially pressed with a 13 mm diameter press mold with a strength of 120 MPa. These pellets were placed on a magnesium oxide plate. To avoid possible contamination by MgO, a layer of the same powder was applied between the MgO plate and the pellets. The pellets were placed in a closed alumina crucible and sintered in air at 1200 °C for 30 h in a high-temperature muffle furnace (Nabertherm GmbH, Lilienthal, Germany). The heating ramp for the calcination and sintering steps was steadily controlled at 5 K·min^−1^ with a natural cooling rate of 5 K·min^−1^ or lower. The densities of the freshly pressed and sintered pellets were determined from their weight and geometry.

### 2.2. Sample Characterization

To obtain information about the phase purity and structure of the samples, characterizations were performed using X-ray diffraction (XRD). The instrument was a D4 Endeavour (Bruker GmbH, Mannheim, Germany) instrument using Cu-Kα radiation and equipped with a 1D detector LYNXEY and a DIFFRAC^plus^ BASIC package, which was released in 2009. All samples were measured from 10 to 60° 2Θ with 0.02° steps. For the measurements, powders and pellets were mortared to fine powder to ensure good statistics. Rietveld refinements were performed for all samples using the program Fullprof [25]. Structural starting models were used from the literature [26,27,28]. The background was fitted using a 6-polynomial function, and the profiles were assumed as asymmetric pseudo-Voigt functions. The lattice parameters were refined, while the atomic positions and thermal parameters were kept according to the literature.

After the sintering process, the samples were polished with SiC sandpaper up to a 4000er grit to remove possible impurities from the surface. For the electrochemical AC impedance spectroscopy (ESI), the polished pellets were covered with a thin layer of gold using a sputter coater (Cressington 108auto Coater, TESCAN GmbH, Dortmund, Germany) for a sputtering time of 150 s. The sputter current was 20 mA. Using a BioLogic VMP-300 Multipotentiostat (Bio-Logic Sciences Instruments Ltd., Claix, France), the impedance spectra of the Al:LLZO samples were measured in Swagelok cells at 25 °C. The frequency was varied from 7 MHz to 1 Hz with an electrical field strength of 10 mV mm^−1^. The pellet dimensions can be seen in Table S1 in the supporting information.

Inductively Coupled Plasma Optical Emission Spectrometry (ICP-OES; Thermo Elemental, IRIS Intrepid iCAP 7600, Waltham, MA, USA) was used to measure the stoichiometry of the sintered Al:LLZO samples by dissolving two 50 mg sample weights in 4 mL sulfuric acid with the addition of 2 g ammonium sulfate under strong heating.

Scanning electron microscopy studies were taken on a Zeiss Supra 50 VP electron microscope (Carl Zeiss Microscopy Deutschland GmbH, Oberkochen, Gernamy) combined with energy dispersive X-ray spectroscopy detector (EDS, X-max 80, Oxford Instruments plc, Abingdon, England) or on a Hitachi TM 3000 tabletop microscope (Hitachi Europe GmbH, Düsselsord, Germany). For microstructural investigations of the sintered specimens, they were embedded in EpoFix epoxy (Struers GmbH, Willich, Germany) and mirror-polished.

The particle size distribution was determined via a laser-scattering method using a laser-scattering particle size distribution analyzer, LA-950V2 (Horiba, Ltd., Kyoto, Japan, distributed by Microtrac Retsch GmbH, Haan, Germany).

Dilatometry experiments were performed on a 402C dilatometer (NETZSCH-Gerätebau GmbH, Selb, Germany).

Thermogravimetric analysis was carried out on a STA449F1 Jupiter calorimeter(NETZSCH-Gerätebau GmbH, Selb, Germany). The experiments were performed in air in a temperature range from 20 to 1200 °C with Al_2_O_3_ sample holder.

## 3. Results

In this study, a substitution of 0.2 mol Al per sum formula was chosen as it results in a fully stabilized cubic phase [13], resulting in a target composition of Li_6.4_Al_0.2_La_3_Zr_2_O_12_. The substitution degree was kept constant to make a correct comparison of the impact of the synthesis routes on the final performance of the material. As mentioned in the Introduction, four different synthesis routes were chosen to assess their potential for industrial upscaling. Next to a classical solid-state reaction (SSR) as a dry synthesis route, three different wet-chemical routes based on an aqueous solution were used: spray-drying (SD), co-precipitation (CP), and a solution-assisted solid-state reaction (SASSR). The solution-assisted solid-state reaction method was first used by Ma et al. for the synthesis of NASICON Na-ion solid electrolyte [29]. We adapted the method for the application of Al:LLZO materials. This synthesis method is similar to the sol-gel method by Pechini but without additional additives, which makes this synthesis route even more viable for an industrial scale.

**Figure 1:**
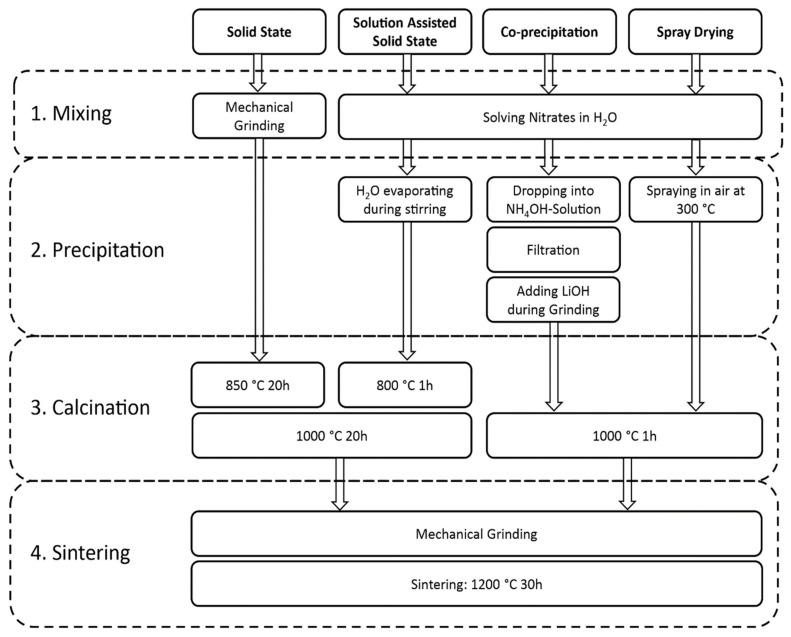
Flowchart showing the major synthesis steps of the four different synthesis methods: solid-state reaction (SSR), solution-assisted solid-state reaction (SASSR), co-precipitation (CP), and spray-drying (SD).

Figure 1 shows a flowchart for the different synthesis methods. All synthesis processes can be divided into four major parts: 1. mixing, 2. precipitation, 3. calcination, and 4. sintering.

The mixing of the precursors for the SSR is completed mechanically with an electrical mortar grinder. A SEM picture of the mixed powder can be seen in Figure S1a in the supporting information. For the wet-chemical methods, the nitrate-based precursors are solved in a nitric acid solution to ensure mixing on an atomic scale.

The precipitation step is omitted for the solid-state reaction but is the major difference in the three wet-chemical methods. In SASSR, the water is slowly evaporated while the aqueous solution is heated at 150 °C and stirred. During the slow evaporation, different stages can be observed, which are similar to a sol-gel reaction: first, the mixture becomes milky, while under further heating and stirring, a gel is formed, which is finally dried. Figure S1b–d shows the as precipitated material for the three different wet chemical synthesis routes. During co-precipitation, the nitric acid solution (without Li) is dropped into a NH_4_OH solution with a pH value of 9.5. Under these conditions, lanthanum, zirconium, and aluminum are precipitated as hydroxides. They were filtrated, dried, and afterward mixed with LiOH in an electrical mortar. The main advantage of this method is that the anionic species are washed out, and therefore different precursors can be used. Thus, cheap precursors, such as halides, which are often produced during the refining of metal ores, can be used. The fastest precipitation occurs during the spray-drying process. Here, the nitrate solution is sprayed into 300 °C hot air as fine droplets that dry immediately, leaving the precipitate residues as hollow spheres, shown in Figure S1d.

**Figure 2:**
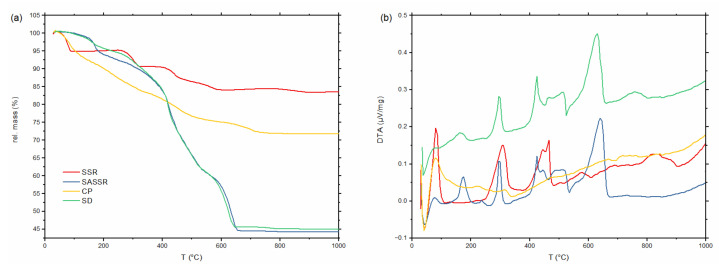
(**a**) Thermogravimetric (TG) and (**b**) differential thermal analysis (DTA) curves for the samples obtained using four different synthesis routes (red—SSR; blue—SASSR; yellow—SD; green—CP). No more mass change can be observed above 900 °C for all samples.

During calcination, the hydroxides, nitrates, and eventual carbonates are thermally decomposed, resulting in the final oxidic specimens. Thermogravimetric curves for the precipitated/mixed precursor materials are compiled in Figure 2. The nitrate species that were precipitated during water evaporation (SASSR and SD) show a very similar thermal decomposition behavior. The mass loss is up to 55% with hardly any change above 650 °C. The hydroxide species (SSR and CP) show a lower mass loss of up to 30%. No further significant loss is observed here above 800 °C. Corresponding DTA measurements are shown in Figure 2b, showing no significant activity above 800 °C except in the SSR sample.

**Figure 3:**
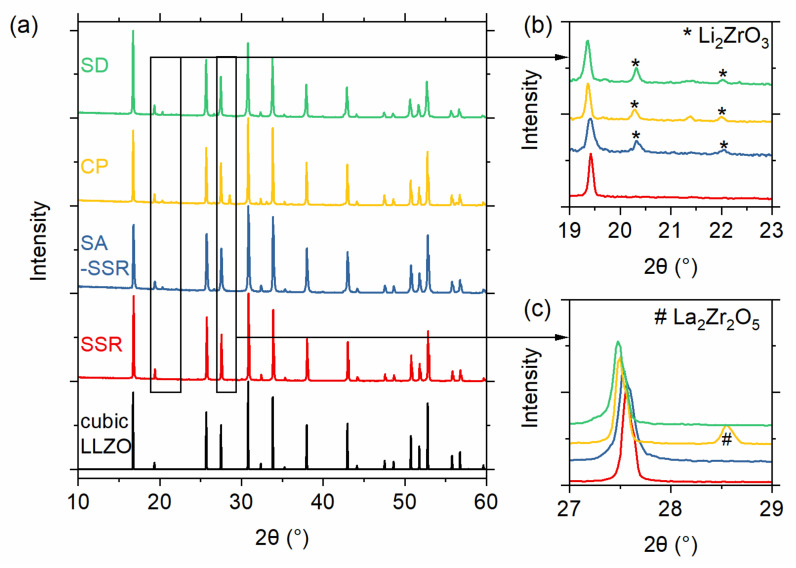
(**a**) XRD pattern of the samples obtained by different synthesis routes (green—SD; blue—CP; red—SASSR; orange—SSR) after the final calcination. The reference pattern for cubic LLZO is shown in black. Small side phases can be observed in the wet-chemical synthesis routes (CP, SASSR, and SD). The 2θ range of (**b**) 19–23° and (**c**) 26–29° is enlarged for better visibility of side phase peaks.

From the TG/DTA measurements, it can be assumed that the calcination is completed at 800 °C for all samples except the SSR sample, where all activities are finished at 900 °C. To assure complete conversion, we chose a slightly higher calcination temperature of 1000 °C, which has been shown to be sufficient in previous works [9,11,30]. The powders from SD and CP synthesis can be directly calcined at 1000 °C for just 1 h to obtain a fully cubic garnet structure. The materials obtained by SSR and SASSR require an additional pre-calcination step at 800 or 850 °C before the final calcination at 1000 °C for 20 h results in a fully cubic garnet. Figure 3a shows the diffraction pattern for all synthesis methods after the final calcination step at 1000 °C. The purest material was achieved by SSR, showing no additional impurity peaks in the pattern. From the lattice parameter of the SSR sample, we calculated the crystallographic density of 5.123 g/cm^3^, which serves as a reference for the density calculations. All wet-chemical routes show minor additional peaks, which can be identified as the Li_2_ZrO_3_ phase, as can be seen in Figure 3b. Additionally, the powder synthesized via co-precipitation shows some additional reflection at 28° and 33° 2Θ, which was identified as pyrochlore-phase La_2_Zr_2_O_5_ (see Figure 3c). ICP-OES results of the material after calcination and sintering are shown in Figure S2 in the supporting information. In particular, the Al content of the sintered CP sample shows a strong deviation from the target value, which could be the reason for the pyrochlore formation. 

The amounts of secondary phases are quite low so that no major effects on the sintering behavior are to be expected. We expect a little effect on the electrochemical performance of the material due to the lower ionic conductivity of Li_2_ZrO_3_ and the insulating nature of the pyrochlore phase La_2_Zr_2_O_5_. To determine the amount of the impurities, Rietveld refinements were performed for each sample. The results of the refinement can be seen in Table S2.

**Figure 4:**
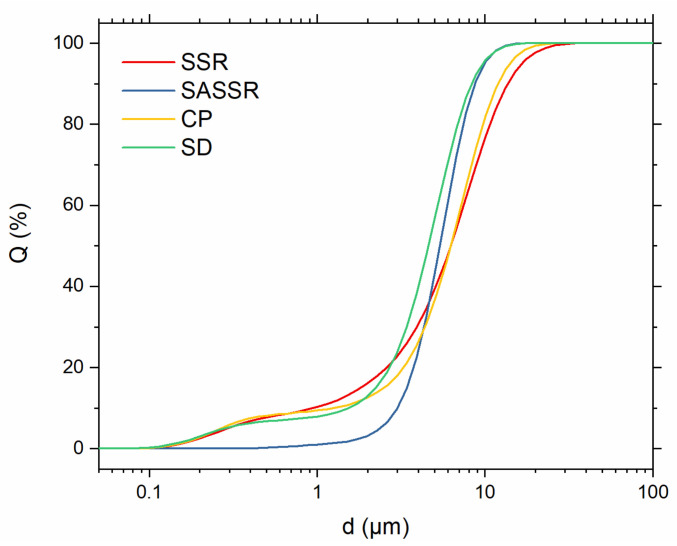
Particle size distributions of the different powders (red—SSR; blue—SASSR; yellow—SD; green—CP). While the SASSR powder shows a quite narrow distribution, the three other powders have a bimodal distribution with one plateau below 1 µm and one between 3 and 10 µm.

Before sintering, the powders were ground using an auto grinder to mill down larger agglomerates and, in the case of SSR, further homogenize the powder. Particle size distributions of the powders before sintering are shown in Figure 4 and Table 2. The particle sizes (d_50_) are quite similar for all powders and range from 4.60 to 6.22 μm. The narrowest distribution was achieved via the SASSR with particle sizes between 3.0 μm (d_10_) and 8.70 μm (d_90_). All other powders show a bilateral distribution, with a smaller d_10_ value of around 1 μm and a larger d_90_ value of 8.34–13.71 μm. The SSR and CP powders have the broadest distribution with the smallest d_10_ and highest d_90_ values for all powders.

**Figure 5:**
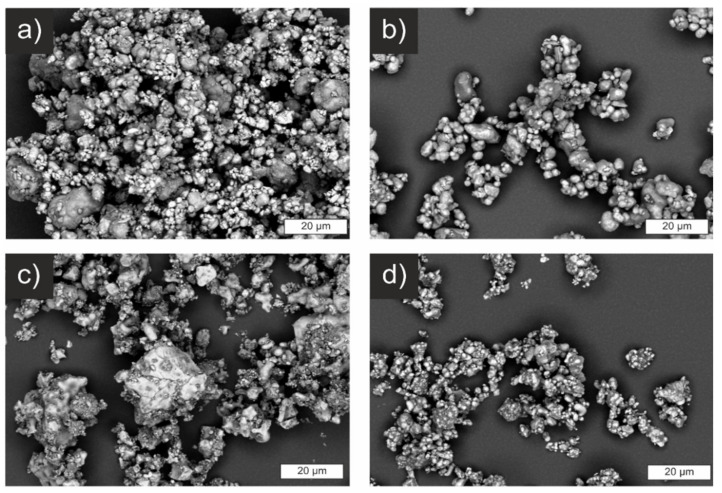
Scanning electron micrographs of samples prepared by different synthesis routes. (**a**) SSR, (**b**) SASSR, (**c**) CP, and (**d**) SD.

Electron micrographs of the powders before sintering are shown in Figure 5. The images strongly confirm the results of the laser-scattering measurements with primary particles in the range of a couple of µm. SSR and CP show larger particles next to smaller ones, while the powder of SASSR is more homogeneous but with generally bigger particles. Especially for the SSR-synthesized powder, larger agglomerates of approx. 50–100 µm are apparent. These were only weakly bound and were easily destroyed during the ultrasonic treatment before the laser-scattering measurement. The only deviation is observed for the SD sample. Similar to the SSR sample, larger agglomerates of smaller particles are visible (Figure 5d). However, the agglomerate size apparent in the micrographs falls within the range measured via light scattering. Thus, they are probably harder and not as easily destroyed by ultrasonic treatment, shifting the measured PSD to higher values. Even though the SD powder already showed the smallest particle size in the laser-scattering measurement, it can be assumed that the de-agglomeration was not complete, and the real particle sizes are even smaller. 

**Figure 6:**
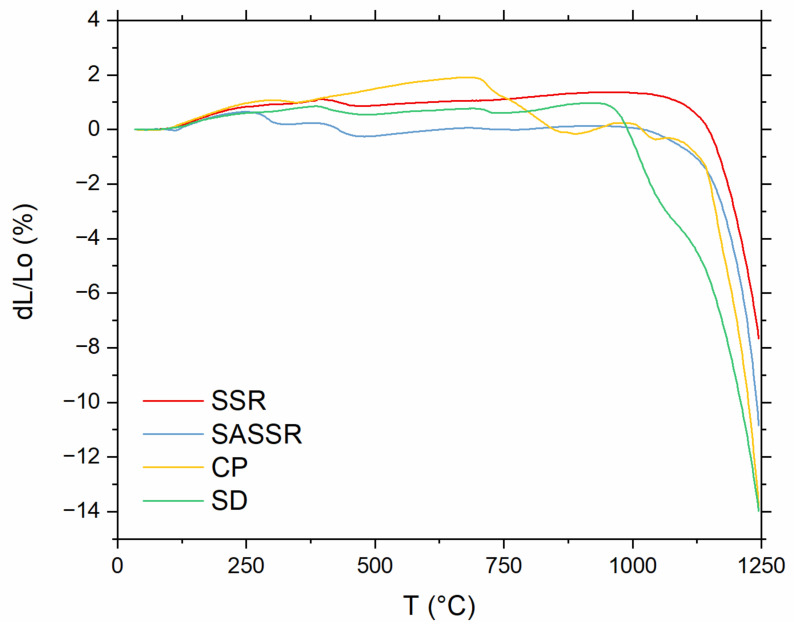
Linear shrinkage measured for green pellets of all four powders (red—SSR; blue—SASSR; yellow—SD; green—CP) between 30 and 1250 °C.

To investigate the sintering behavior, dilatometry measurements for all four powders were performed and are compiled in Figure 6. The densification of the powders produced by SSR and SASSR starts at around 1100 °C and shows a very similar shrinkage behavior. The powder from CP shows some shrinkage around 700 °C. Together with the XRD data, one could suggest that the calcinated powder is not fully converted yet, and a chemical reaction towards LLZO can be observed here. The actual onset of densification is, similar to SSR and SASSR, at 1100 °C. Again, the SD powder shows a different trend. Shrinkage already starts at 1000 °C, which is 100 °C lower than for the other three. Most likely, this behavior is due to the much smaller primary particle size obtained in the spray-drying process, which results in higher sintering activity [31].

To keep the conditions similar for all samples, the sintering temperature was set at 1200 °C. At this temperature, all powders are sintering and shrinking. The sintering time was set to 30 h to ensure the highest density for all powders. By excluding the impact of sintering time and temperature, our samples only show differences in relative density of 90 ± 3%, making them ideal for further electrochemical analysis. In an industrially scaled process, the sintering temperature and time would, of course, be adapted to obtain the required density, component performance, and cost target.

**Figure 7:**
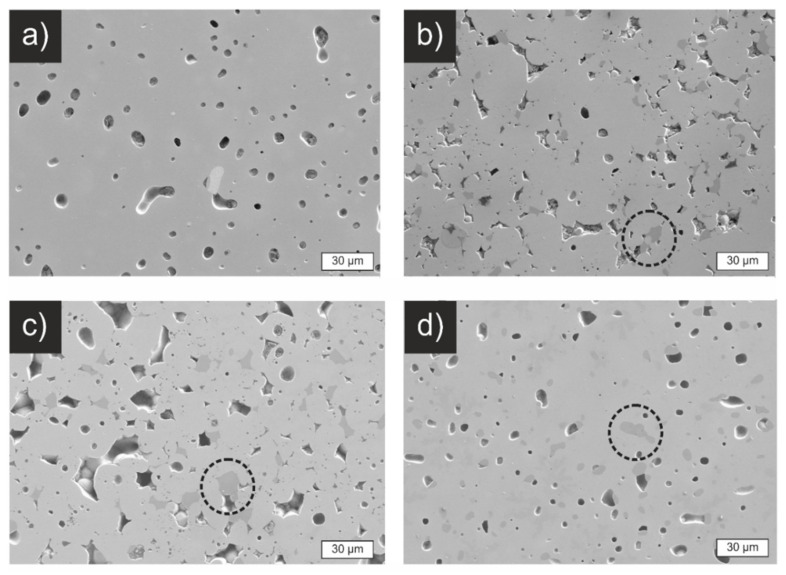
Scanning electron micrographs of cross-sections of polished, sintered pellets. (**a**) SSR, (**b**) SASSR, (**c**) CP, (**d**) SD. The pellets of SSR and SD show dense pellets with closed porosity, while SASSR and CP have a more open porosity, which is reflected in the lower relative density of just 87%. Impurities of Li_2_ZrO_3_ in the samples SASSR, CP, and SD are visible in slightly darker areas and marked with dashed circles.

Figure 7 shows polished cross-sections of sintered pellets from the four different materials. The SSR pellet (Figure 7a) shows 93% relative density and features a closed porosity with pore sizes of around 10 µm. The pellets from SASSR and CP show a higher, more open porosity, as can be seen in Figure 7b,c. This is also reflected in a lower relative density, around 87% (Table 3). They also show darker areas that can be explained by the secondary Li_2_ZrO_3_ phase that is also observable in the XRD pattern (Figure S3 in the supporting information). The cross-section of the SD sample is again similar to the SSR with closed porosity and a relative density of 93%. Since the same morphology is observed for the SD and SSR samples, an effect of the minor Li_2_ZrO_3_ phase on the sintering behavior can be excluded.

The XRD pattern (Figure S3) of crushed and ground pellets shows that the cubic garnet phase is not influenced by the rather long sintering procedure. The wet-chemical synthesis routes still show the small impurity of Li_2_ZrO_3_, which was already observed in the calcined material. The CP sample shows a shoulder, indicating a splitting into the tetragonal garnet phase, but the small pyrochlore phase observed in the calcined powder is not visible any longer. This confirms the assumption that a chemical reaction takes place between the pyrochlore phase and the excess LiOH during the sintering process, producing cubic LLZO. A similar reaction was previously observed in LLZO produced via flame-spray pyrolysis [32] and could also contribute to the unusual shrinkage behavior of this material observed in TG/DTA (Figure 6).

**Figure 8:**
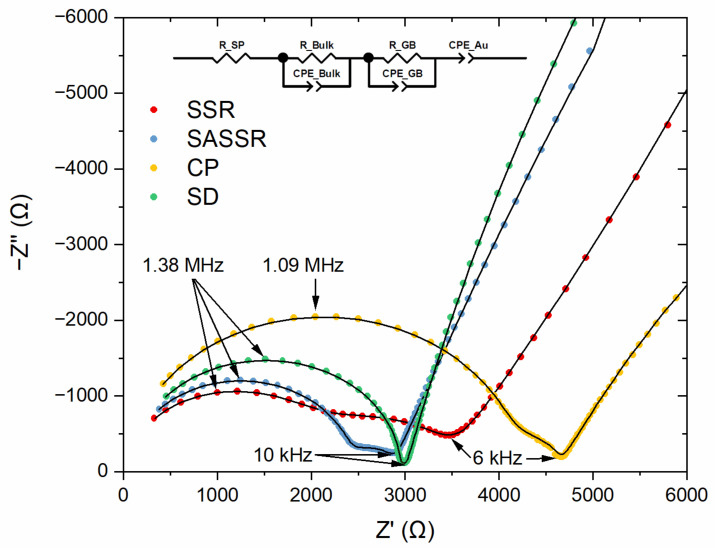
Nyquist plots and equivalent circuit fits of the impedance spectra for the samples of all four different synthesis routes (red—SSR; blue—SASSR; yellow—SD; green—CP).

The electrical properties of the samples were investigated using impedance spectroscopy. Figure 8 shows the Nyquist plots and the fits of the impedance spectra for all samples. The spectra show two contributing semicircles in the high-frequency range and a capacitive tail in the low-frequency region. The semicircles correspond to the bulk resistance and the grain boundary resistance of LLZO and can be described with two R-CPE elements. For the samples with side phases, an additional resistor (*R*_SP_) was necessary for a good description. The low-frequency tail is a clear sign for ion-blocking electrodes, typical for gold electrodes, and may be described, for example, by a CPE element, as reported before [4,9].

In order to assign the R-CPE elements to the corresponding physical counterparts, the effective capacitance *C* was calculated from the fitted resistance *R*, CPE coefficient *Q*, and the exponential parameter α (Equation (1)). [33,34] α describes the non-homogeneity in the system. For example, a rough or porous surface can cause a double-layer capacitance to appear as a constant phase element with an α value between 0.9 and 1. The case α = 1 describes an ideal capacitor, while the case α = 0 describes a pure resistor. For the mentioned capacitance calculation, α should be at least 0.75. The fitted resistances and effective capacitances can be found in Table 4.

(1) $$C=\frac{{\left(Q\cdot R\right)}^{\frac{1}{\alpha }}}{R}$$

The capacitances fit quite well to the reported values for the bulk (10^−11^ F) and the grain boundaries (10^−7^–10^−9^ F) of an ion conductor and are in the same regime as shown for Al:LLZO samples with similar Al concentrations in literature before [35,36]. However, the lower capacitance combined with higher resistance (SSR) and the higher capacitance with lower resistance (SD) indicate a larger and smaller amount of grain boundaries, respectively. This is due to the grain sizes in the sintered materials [37] and shows a better grain growth for wet-chemical routes. All resistances and the pellet geometry (L, S) were used to calculate the total ionic conductivity σ_total_, respectively; only *R*_Bulk_ was used to calculate the bulk conductivity σ_Bulk_ of LLZO (Equation (2)). The conductivity of the grain boundaries was calculated from the pellet geometry, the fitted grain boundary resistance, and the ratio of bulk and grain boundary capacitance (Equation (3)) [38,39]. The calculated conductivities can be found in Table 4.

(2) $$\sigma_{\mathrm{total}}=\frac{L}{SR_{\mathrm{total}}}$$

(3) $$\sigma_{\mathrm{GB}}=\frac{L}{SR_{\mathrm{GB}}}\cdot \frac{C_{\mathrm{Bulk}}}{C_{\mathrm{GB}}}$$

The total conductivities mirror the density and the purity of the materials, the highest σ_total_ being observed in the SD sample (3.28(3) × 10^−4^ S/cm), followed by SSR (3.14(7) × 10^−4^ S/cm), which are also the two samples with the highest observed density. The CP sample shows the lowest σ_total_ (2.02(2) × 10^−4^ S/cm), and SASSR has a mediocre σ_total_ of 2.64(3) × 10^−4^ S/cm. The phase purity shows its influence on the bulk conductivities of the materials. While the pure SSR product shows bulk conductivity of 5.19(7) × 10^−4^ S/cm, the materials with only Li_2_ZrO_3_ contamination show just ~64% of this conductivity. It becomes even further reduced to 44% if the tetragonal LLZO phase is present. As can be seen in the SEM images, the side phases form grains within the LLZO phase and therefore lower the conductivity due to an effective prolonged path for lithium ions. The grain boundary conductivities of all routes are quite similar and typically one to two orders of magnitude lower than the bulk conductivity (σ_GB_ ≈ 10^−5^–10^−6^ S/cm). However, the large errors also reveal the limitations of the calculation method, and this calculation is only used to calculate the order of magnitude of σ_GB_.

In total, the impedance spectra show that the atomic mixing of the wet-chemical routes results in clean interfaces between the grains and supports grain growth, while the SSR route seems to have higher grain boundary resistances due to diffusion-controlled chemical processes in precursor particles.

## 4. Discussion

This discussion will focus on connecting the obtained material parameters, such as phase purity, particle size, sintering behavior, density, and conductivity, to a cost-sensitivity analysis for industrial production of Al:LLZO via the four synthesis routes. Even though a specific price estimate is only possible for industrial material manufacturers, we are still able to identify possible cost advantages (or at least sensitivities) of the used synthesis methods with respect to precursor price, effective workload, scale-up potential, and calcination time. Unfortunately, we are not able to assess the initial investment cost for equipment, although it is an important factor for industrial production.

To enable this comparison, we show, in the Results section, that all material parameters were similar after sintering. Table 5 summarizes the main results for a better discussion.

If performed correctly, all four synthesis methods yield the same high-quality powder in terms of material parameters. In each case, cubic Al:LLZO with a purity of at least 93% and total Li-ion conductivity of at least 2 × 10^−4^ S/cm was achieved. The fact that the electrochemical performance is relatively independent of the synthesis route is a result of the sintering conditions we chose, which were specifically selected to demonstrate that the desired properties can be achieved by all four routes, which allows a cost-sensitivity analysis. 

For an industrial production process of all-solid-state batteries, the particle size distribution will also be highly relevant since it has a major impact on the sintering behavior in the component-manufacturing step. Unfortunately, since we cannot analyze all possible component manufacturing processes and requirements, the impact of the particle size obtained in synthesis and its resulting impact on sintering behavior needs to be omitted from the cost-sensitivity analysis.

Nevertheless, an evaluation of the scaling potential of each synthesis route is possible, using five synthesis parameters that have the highest impacts on the price of the final material:

1. Precursor price—raw material cost;
2. Calcination time (as a measure of energy cost);
3. Scale—initial investment and output (economy of scale);
4. Workload—personnel costs;
5. Material performance.

The parameters given here are experimental results from our laboratory-scale production. It is important to keep in mind that when upscaling to large-scale industrial production, parameters such as workload and scale may differ drastically from those of laboratory production.

### 4.1. Precursor Price

The main advantage of the solid-state reaction is that relatively cheap oxidic precursors can be used. In comparison to this, the wet-chemical synthesis methods require much more expensive solvable species. In our case, we used nitrate species, but in general, it should be possible to use all soluble and flammable compounds, such as organic materials. Here, co-precipitation has another advantage. Since its anionic species are not burned out like in SD and SASSR but washed out during the filtration step, cheaper, non-combustible species such as halides might also be suitable. The prices for the precursors will drop dramatically when large quantities of the material are needed. However, the ratios between the different kinds of materials will stay similar. For example, will nitrates always be more expensive than oxides?

### 4.2. Calcination Time

At 40 h, the SSR requires, by far, the longest calcination time. The educts are physically mixed and ground. The grains must react with each other, and the final product is formed by the relatively slow solid-state diffusion. It is reduced to 21 h for SASSR due to the atomical premixing in the solution. However, since it is dried by a relatively slow water-evaporation process, not all reactants precipitate at the same time, and segregation occurs to some extent. A longer calcination step is still required. This demixing is avoided in the CP and SD processes. Here, the precipitated product is mixed at the atomic level, no slow diffusion is required, and the calcination time is reduced to only 1 h.

Long calcination times at high temperatures are crucial within continuous processes, which are required for large-scale industrial production. Not only does the energy cost drop by reducing the calcination time, but the investment price also drops, since smaller furnaces are sufficient for shorter calcination times.

### 4.3. Scale

We are in a comfortable position to have a pilot plant-sized spray dryer in our institute, which can produce up to 1 kg Al:LLZO per hour. Therefore, scaling up this synthesis into a kg scale was rather easy. The other routes were performed on a normal lab scale between 0.05 and 0.1 kg. As described in the introduction, all of these methods are saleable and already established in the industry on large scales for other materials. Thus, we set the scale to max (kg range) for all four methods to distinguish them from experimental methods such as nebulized spray pyrolysis, etc., which are not easily scalable and produce only mg amounts.

### 4.4. Workload

Again, the presence of the pilot plant spray dryer reduces the workload drastically in comparison to the other methods. The solid-state reaction is pretty much straightforward, requiring more equipment but keeping the effective workload relatively low. In addition, the solid precursors are easier to handle and require less attention from the manufacturer. In contrast, SASSR, and especially CP, require quite a large amount of effort in the lab. This might change on an industrial scale but, in our opinion, follows a similar overall trend.

### 4.5. Material Performance

In our process, we chose long and high sintering conditions to omit the differences between the powders (particle size, sinterability) to obtain a maximal high-performing LLZO pellet. Still, there are small differences between the sintered pellets. While the total conductivity is very similar, there are big differences in the grain boundary resistance, which is significantly lower for the wet-chemical routes in comparison to the SSR. The density is around 90% for all samples and the highest for SSR and SD. The phase purity was high for all samples, but the CP route, in particular, produced an unwanted pyrochlore phase, which is why it ranks slightly lower than the other three. However, we are sure that with an optimized process design, the side phases can be avoided. To compare material performance with the cost-determining parameters, we chose conductivity and purity for comparison.

**Figure 9:**
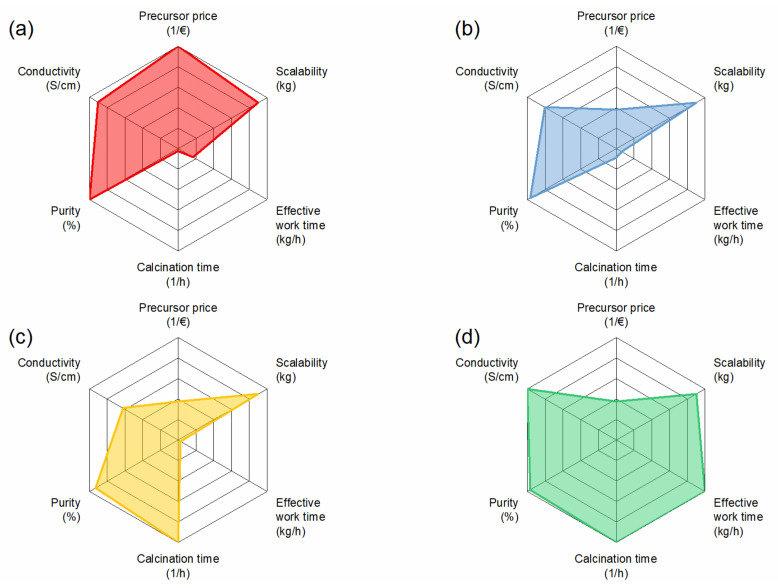
Radar plots of selected material and cost parameters. All parameters were normalized to 1 for a better comparison. Since all synthesis methods are scalable, we put the value for all methods to 0.9. For precursor price and calcination time, the inverse values are shown. (**a**) SSR, (**b**) SASSR, (**c**) CP, and (**d**) SD.

For better visualization of the evaluation, the parameters were plotted in a radar plot for each synthesis, as can be seen in Figure 9. From the results, the solution-assisted solid-state reaction has the lowest potential for low-cost production of Al:LLZO due to long calcination times and only medium precursors prices. The solid-state reaction scores particularly well in terms of precursor price, which makes it attractive for scaling up, but it also has disadvantages in terms of calcination time, which leads to high energy costs. Co-precipitation has a high potential as it requires low calcination times and allows the use of cheaper precursors, but purity needs to be tightly controlled to achieve optimal performance. Overall, the highest potential for low-cost production of Al:LLZO was determined as the spray-drying route, with the lowest calcination time and workload, compensating for the medium precursor prices.

## 5. Conclusions

We have shown that solid-state reaction, solution-assisted solid-state reaction, co-precipitation, and spray-drying are suitable methods to obtain aluminum-substituted LLZO with high Li-ion conductivity. The total conductivities of the sintered samples are similar for all synthesis methods and in the range of 2.0–3.3 × 10^−4^ S/cm. Thus, all methods can be used to reproduce the results in academic research. For industrial synthesis, further parameters such as the precursors’ price, calcination time, and effective workload will determine the most cost-effective method. The most promising methods for upscaling to industrial levels are spray-drying and co-precipitation, which minimize the required calcination time to only 1 h at 1000 °C and consequently reduce energy costs. While spray-drying was the most effective in terms of labor hours/kg and energy costs, the solid-state reaction from oxides and co-precipitation could be advantageous in terms of cheaper precursors.

## Figures and Tables

**Table 1:** Selected literature data to different synthesis methods of Al:LLZO.

Stoichiometry	Synthesis	Total Conductivity	Ref.
Li_7_La_3_Zr_2_O_12_ (0.204 mol Al)	Solid state	4 × 10^−4^ (RT)	[13]
Li_6.4_Al_0.2_La_3_Zr_2_O_12_	Solid state	3.4 × 10^−4^ (RT)	[11]
Li_7_La_3_Zr_2_O_12_ (28 mol% Al)	Solid state	3.5 × 10^−4^	[19]
Li_6.16_Al_0.28_La_3_Zr_2_O_12_	Sol-Gel	1.1 × 10^−4^ (33 °C)	[20]
Li_7_La_3_Zr_2_O_12_ (1.2 wt.% Al)	Sol-Gel	2 × 10^−4^	[21]
Li_7_La_3_Zr_2_O_12_ (Al = 0–0.25)	Nebulized spray pyrolysis	4.4 × 10^−6^	[22]
Li_6.4_Al_0.2_La_3_Zr_2_O_12_	Combustion	5.1 × 10^−4^ (30 °C)	[24]
Li_6.25_Al_0.25_La_3_Zr_2_O_12_	Co-precipitation	3.2 × 10^−6^ (30 °C)	[23]

**Table 2:** Parameters of the particle size distribution for the different samples determined by laser-scattering method.

Sample	d_10_	d_50_	d_90_
SSR	0.94	6.22	13.71
SASSR	3.00	5.38	8.70
CP	1.25	6.23	12.00
SD	1.54	4.60	8.34

**Table 3:** Density of green and sintered samples prepared by different synthesis routes.

Parameter	SSR	SASSR	CP	SD
Green pellet density (g/cm^3^)	2.910	2.977	3.138	3.039
Rel. green density (%)	56.7	58.0	61.1	69.6
Density of sintered pellet (g/cm^3^)	4.767	4.444	4.445	4.747
Rel. sintered density (%)	93.1	86.8	86.8	92.7

**Table 4:** Fitted resistances, calculated capacitances, and the total, bulk, and grain boundary conductivities for the different samples.

Parameter	SSR	SASSR	CP	SD
*R*_SP_ (Ω)	0	51.5(12)	50(4)	86(4)
*R*_Bulk_ (Ω)	2018(16)	2364(2)	4106(9)	2859(4)
*C*_Bulk_ (10^−11^ F)	6.1(4)	4.819(6)	3.65(16)	4.03(13)
*R*_GB_ (Ω)	1318(31)	412(4)	464(6)	41(3)
*C*_GB_ (10^−8^ F)	0.16(5)	0.95(15)	0.70(12)	12.37(13)
σ_total_ (10^−4^ S/cm)	3.14(7)	2.64(3)	2.02(2)	3.28(3)
σ_Bulk_ (10^−4^ S/cm)	5.19(7)	3.19(3)	2.28(2)	3.44(3)
σ_GB_ (10^−6^ S/cm)	30(12)	8.7(15)	10.5(10)	6.3(12)

**Table 5:** Summary of the main material parameters as described in Discussion.

Sample	Phase Purity (%)	Average Particle Size (µm)	Sintering Onset Temperature (°C)	Rel. Density (%)	σ_total_ (10^−4^ S/cm)
SSR	100	6.22	1100	93.1	3.14(7)
SASSR	97	5.38	1100	86.8	2.64(3)
CP	93	6.23	1000	86.8	2.02(2)
SD	97	4.60	1000	92.7	3.28(3)

## Data Availability

The data presented in this study are available upon request from the corresponding author.

## Supplementary Materials

The following are available online at https://www.mdpi.com/article/10.3390/ma14226809/s1, Figure S1: SEM pictures of (a) mixed precursors for SSR, (b), (c) and (d) show the precipitated materials of SASSR, CP and SD, respectively, Figure S2: ICP-OES results of differnet synthesis routes after calcination and after sintering., Figure S3: XRD pattern of the different synthesis routes (green SD; yellow CP; blue SASSR; red SSR) after sintering. Figure S4: Particle Size distributions by laser scattering of the different powder samples: (a) SSR, (b) SASSR, (c) CP, (d) SD., Table S1: Pellet mass and dimensions of the different sintered samples., Table S2: Results of the Rietveld refinements of the calcined powders.
