# Supplementary material for: Evaluation of Scalable Synthesis Methods for Aluminum-Substituted Li7La3Zr2O12 Solid Electrolytes
Source: Materials (Basel). 2021 Nov 11;14(22):6809. doi: 10.3390/ma14226809 (PMC8620275; doi:10.3390/ma14226809)
Supplement: Supplementary file 1 [file materials-14-06809-s001.zip › materials-1426329-supplementary.pdf]

materials

Supplementary Material

Evaluation of Scalable Synthesis Methods for Aluminum-Substituted $Li_7La_3Zr_2O_{12}$ Solid Electrolytes

Markus Mann [1], Michael Küpers [1], Grit Häuschen [1], Martin Finsterbusch [1,2,*], Dina Fattakhova-Rohlfing [1,2] and Olivier Guillon [1,2,3]

[1] Institute of Energy and Climate Research (IEK-1) Materials Synthesis and Processing, Forschungszentrum Jülich GmbH, Wilhelm-Johnen-Strasse, 52425 Jülich, Germany; m.mann@fz-juelich.de (M.M.); m.kuepers@fz-juelich.de (M.K.); g.haeuschen@fz-juelich.de (G.H.); d.fattakhova@fz-juelich.de (D.F.-R.); o.guillon@fz-juelich.de (O.G.)

[2] Helmholtz-Institut Münster (IEK-12), Forschungszentrum Jülich GmbH, Corrensstraße 46, 48149 Münster, Germany

[3] Jülich Aachen Research Alliance, JARA-Energy, 52425 Jülich, Germany

* Correspondence: m.finsterbusch@fz-juelich.de

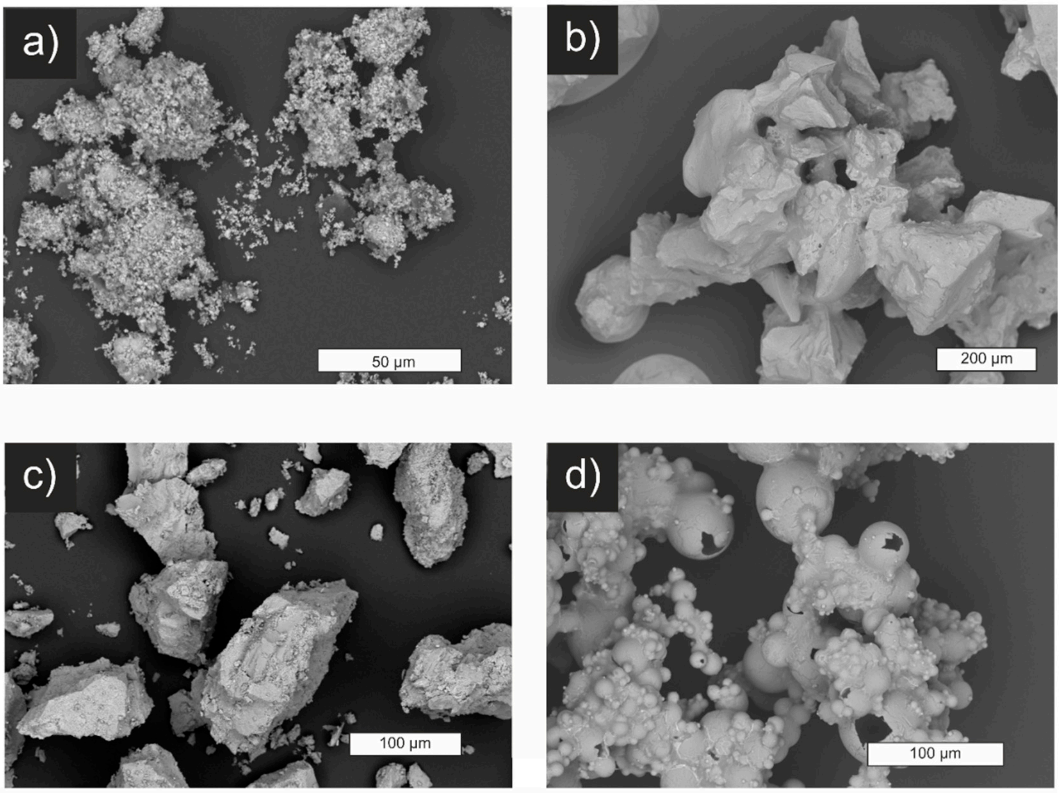

Figure S1. SEM pictures of (a) mixed precursors for SSR, (b), (c) and (d) show the precipitated materials of SASSR, CP and SD, respectively.

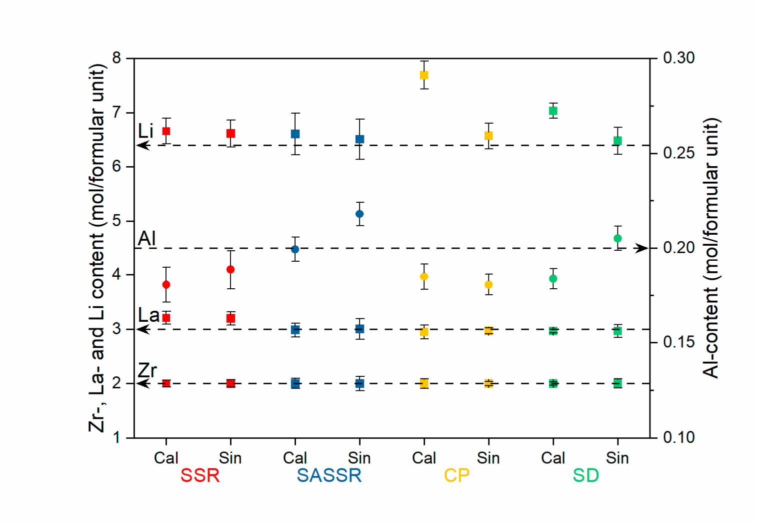

Figure S2. ICP-OES results of differnet synthesis routes after calcination and after sintering.

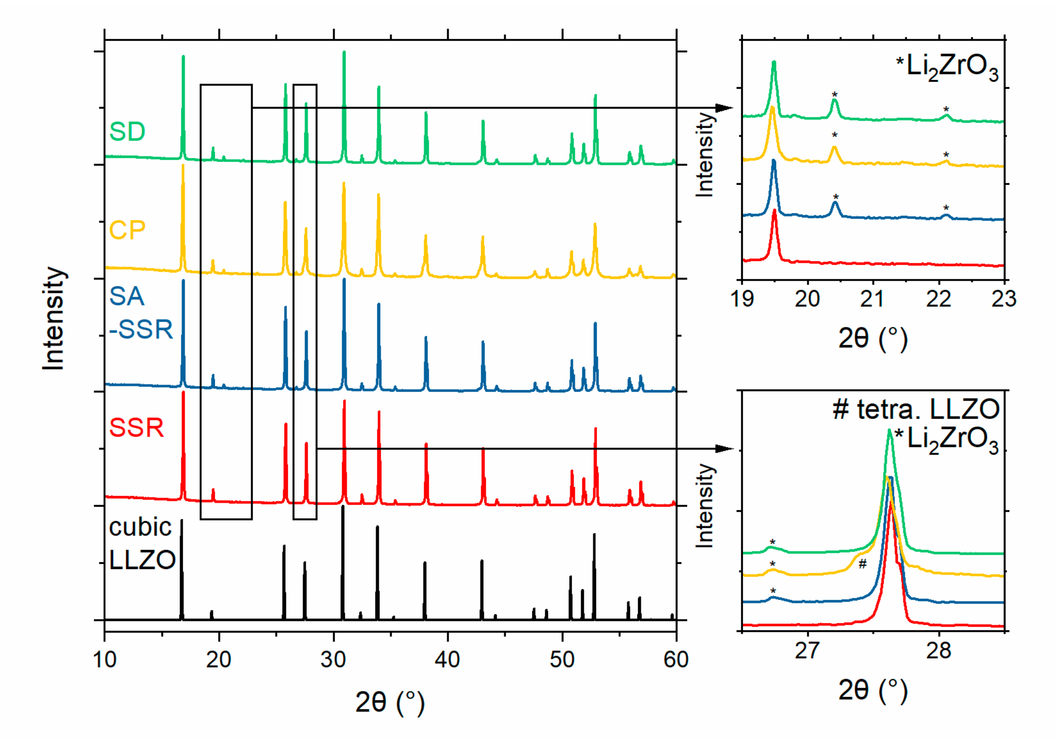

Figure S3. XRD pattern of the different synthesis routes (green SD; yellow CP; blue SASSR; red SSR) after sintering. The reference pattern for cubic LLZ is shown in black. Small side phases can be observed in the wet chemical synthesis routes (CP, SASSR and SD) as can be seen in the right inlays.

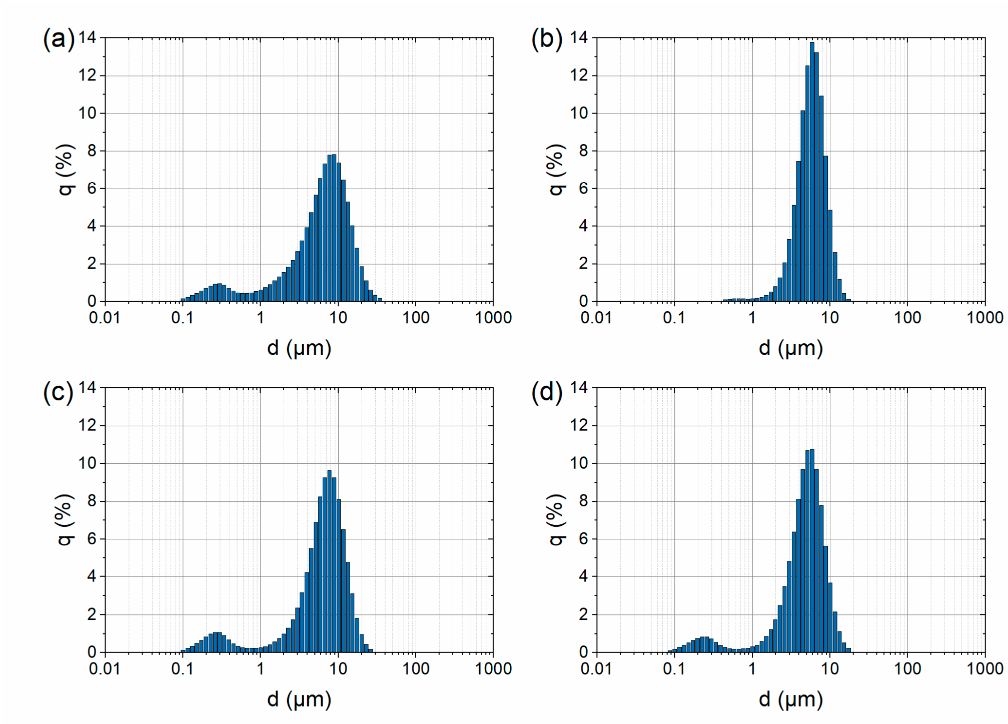

Figure S4. Particle Size distributions by laser scattering of the different powder samples: (a) SSR, (b) SASSR, (c) CP, (d) SD.

Table S1. Pellet mass and dimensions of the different sintered samples.

Parameter	SSR	SASSR	CP	SD
m (g)	5.6116	3.3589	4.1654	4.1517
h (mm)	11.1	7.55	9.36	9.27
d (mm)	11.62	11.29	11.29	10.96

Table S2. Results of the Rietveld refinements of the calcined powders.

Parameter	SSR	SASSR	CP	SD
cubic garnet phase (%)	100	97(1)	93(1)	97(1)
Lattice parameter *a* (Å)	12.968(1)	12.977(1)	12.972(1)	12.997(1)
R_{Bragg}; R_f	4.81; 4.10	10.9; 7.75	11.0; 6.06	3.73; 2.62
Li_2ZrO_3 (%)	-	3(1)	3(1)	3(1)
R_{Bragg}; R_f	-	23.4; 29.0	14.4; 15.4	18.6; 10.7
$La_2Zr_2O_5$ (%)	-	-	4(1)	-
R_{Bragg}; R_f	-	-	10.0; 9.23	-
